# Exploring the Predictive Power of News and Neural Machine Learning Models for Economic Forecasting

**DOI:** 10.1007/978-3-030-66981-2_11

**Published:** 2021-01-15

**Authors:** Luca Barbaglia, Sergio Consoli, Sebastiano Manzan

**Affiliations:** 8 UniCredit, Milan, Italy; 9 UniCredit, Rome, Italy; 10 UniCredit, Milan, Italy; 11 UniCredit, Rome, Italy; 12 ENEA Portici Research Center, Portici, Italy; 13 UniCredit, Rome, Italy; European Commission, Joint Research Centre, Directorate A-Strategy, Work Programme and Resources, Scientific Development Unit, Via E. Fermi 2749, 21027 Ispra, VA Italy

**Keywords:** Economic and financial forecasting, Neural time series forecasting, Deep learning, Recurrent neural networks, Long short-term memory networks, Word embedding, News analysis

## Abstract

Forecasting economic and financial variables is a challenging task for several reasons, such as the low signal-to-noise ratio, regime changes, and the effect of volatility among others. A recent trend is to extract information from news as an additional source to forecast economic activity and financial variables. The goal is to evaluate if news can improve forecasts from standard methods that usually are not well-specified and have poor out-of-sample performance. In a currently on-going project, our goal is to combine a richer information set that includes news with a state-of-the-art machine learning model. In particular, we leverage on two recent advances in Data Science, specifically on Word Embedding and Deep Learning models, which have recently attracted extensive attention in many scientific fields. We believe that by combining the two methodologies, effective solutions can be built to improve the prediction accuracy for economic and financial time series. In this preliminary contribution, we provide an overview of the methodology under development and some initial empirical findings. The forecasting model is based on DeepAR, an auto-regressive probabilistic Recurrent Neural Network model, that is combined with GloVe Word Embeddings extracted from economic news. The target variable is the spread between the US 10-Year Treasury Constant Maturity and the 3-Month Treasury Constant Maturity (T10Y3M). The DeepAR model is trained on a large number of related GloVe Word Embedding time series, and employed to produce point and density forecasts.

## Introduction

Monitoring the current and forecasting the future state of the economy is of fundamental importance for governments, international organizations, and central banks. Policy makers require timely macroeconomic information in order to design effective policies that can foster economic growth and preserve societal well-being. However, they rely on economic indicators produced by statistical agencies that are released at low frequencies (e.g., monthly or quarterly), with considerable delays, and that are often subject to substantial revisions. With such an incomplete information set in real-time, the forecasts produced by economists are very uncertain and inaccurate even when forecasting the current economic situation as well as the future, thus making the task extremely challenging. Moreover, in a global interconnected world, shocks and changes originating in one economy could move quickly to other economies affecting productivity levels, job creation and welfare in different geographic areas. However, greater interdependence also means that the current and future conditions of a market are linked to instabilities and extreme events originated abroad. All these factors make the economic forecasting task extremely difficult, both in the short and in the medium-long run.

In this context, economists and researchers can leverage on the rapid advances in information and communications technology experienced in the last two decades, which have produced an explosive growth in the amount of information available leading to the era of *Big Data* [17]. Novel and alternative data sets can potentially contribute greatly to help us in monitoring and forecasting economic activity given its timeliness and its economic relevance. A major source of such information is represented by news text, since it discusses important events, economic and financial news releases, and experts opinions, among others, that can serve as a basis for economic and financial decisions [5]. In addition, news affect consumers’ perception of the economy through three channels. First, the news media convey the latest economic data and professionals’ opinion to consumers. Second, consumers receive a signal about the economy through the tone and volume of economic reporting. Last, the greater the volume of news about the economy, the greater the likelihood that consumers will update their expectations about the economy [7].

In a currently on-going research project, we aim to explore the predictive power of news for forecasting of economic and financial time series by leveraging on the recent advances in Data Science, specifically on Word Embedding [6, 18, 20] and Deep Learning [3, 15, 21, 22] models. Word Embedding models represent the contextual information of a given corpora and capture syntactic and semantic information with respect to the data set used for building the embeddings. Following a specific NLP information extraction pipeline, which we describe in Sect. 4, we extract sentences referring to a specific economic aspect from the news media. Then, from those sentences, we derive signals corresponding to the Word Embedding of the extracted sentences. For example, if we deal with daily economic time series data, the Word Embedding signals are calculated on the sentences extracted each day from the news text. In our on-going research activity we aim to compare the predicting capabilities of the most popular Word Embedding methods, from the widely used Word2Vec [18] and GloVe [20] models, to the most recent context-dependent models like BERT [6] or GPT-3 [4]. In this contribution we only consider Glove embeddings. The goal is to extract the hidden information embedded in economic news to provide useful predictive signals that can be used as additional features to improve the accuracy of economic forecasts [9].

In addition, we use a novel forecasting model based on Deep Learning [3, 14, 15] for addressing the economic forecasting task. In particular, in this work we rely on DeepAR [22], a powerful neural forecasting methodology that produces accurate probabilistic forecasts, based on training an auto-regressive Recurrent Neural Network (RNN) model on a large number of related time series, which in our case are the Word Embedding signals. We believe that by combining these two strong Data Science methodologies, that is Word Embedding and Deep Learning, effective solutions can be built to improve the accuracy of prediction tasks for economic and financial time series.

In this contribution we provide an overview of the methodology under development. We report on some preliminary findings on the use-case application of DeepAR along the Word Embedding extracted by a GloVe pre-trained model from United States (US) news ranging from January 1982 to September 2019, with the goal of predicting the future values of the US 10-Year Treasury Constant Maturity Minus 3-Month Treasury Constant Maturity (T10Y3M) time series given its past values.

## Background

Information encoded in text is a rich complement to the more structured kinds of data traditionally used in empirical research [10]. In recent years, we have seen an intense use of textual data in different areas of research. The idea consists of transforming strings of raw text into numeric variables, and then use them as predictors in different models. News articles, in particular, represent a relevant data source to model economic and financial variables, and several studies have already explored this additional source of information. For a recent overview on the application of text analysis in economics and finance the reader is also referred to [1, 10].

On the other end, there is a vast literature on the use of Deep Learning in the context of time series forecasting, e.g. see [14, 21]. For a survey the reader is referred to [3, 13, 15]. Neural Networks (NNs) in forecasting have been typically applied to individual time series, i.e. a different model is fitted to each time series independently [23]. Although it is fairly straightforward to use classic Multilayer Perceptrons NNs (aka MLPs) on large data sets, its use on medium-sized time series is more difficult due to the high risk of over-fitting. Classical MLPs can be adapted to address the sequential nature of the data by treating time as an explicit part of the input. However, such an approach has some inherent difficulties, namely the inability to process sequences of varying length and to detect time invariant patterns in the data. A more direct approach is to use recurrent connections that connect the neural networks hidden units back to themselves with a time delay. This is the principle at the base of Recurrent Neural Networks (RNNs) [15, 21], which are NNs specifically designed to handle sequential data that arise in applications such as time series, natural language processing and speech recognition. In finance, for example, the authors in [16] developed a multi-task RNN with high-order Markov random fields to predict stock price movement direction based upon a single stock’s historical records together with its correlated stocks.

Although RNNs have been widely used in practice, it turns out that training them is quite difficult given that they are typically applied to very long sequences of data. A common issue while training very deep neural networks by gradient-based methods using back-propagation is that of vanishing or exploding gradients which renders learning impossible. Long Short-Term Memory Networks (LSTMs) were proposed [12, 14] to address this problem. Instead of using a simple network at each time step, LSTMs use a more complicated architecture composed of a cell and gates which control the flow of input to the cell as well as decide what information should be kept inside the cell and what should be propagated to the next time step [14]. The cell has a memory state which is propagated across time along with the output of the LSTM unit, which is itself a function of the cell state. Unlike the output of the LSTM unit, the cell state undergoes minimal changes across time, thus the derivative with respect to the cell state does not decay or grow exponentially [11]. Consequently, there is at least one path where the gradient does not vanish or explode making LSTMs suitable for processing long sequences. For details on the working mechanisms behind RNNs and LSTMs we suggest the reader to go through the online tutorial in [19].

Recently, in [22] the authors have proposed DeepAR, an RNN-based forecasting model using LSTM or GRU cells, the latter being a simplification of LSTMs that do not use a separate memory cell and may result in good performance for certain applications. At each time step, DeepAR takes as input the previous time points and covariates, and estimates the distribution of the value of the next period. This is done via the estimation of the parameters of a pre-selected parametric distribution (e.g. negative binomial, student *t*, gaussian, etc.)^1^. Training and prediction follow the general approach for auto-regressive models [22]. One feature makes this forecasting setting appealing: in probabilistic forecasting one is interested in the full predictive distribution, not just a single best realization, making the analysis more robust and reducing uncertainty in the downstream decision-making flow. In addition to providing more accurate forecasts, DeepAR has also other advantages compared to classical approaches and other global methods [22]: (i) As the model learns seasonal behavior and dependencies on given covariates across time series, manual feature engineering is drastically minimized; (ii) DeepAR makes probabilistic forecasts in the form of Monte Carlo samples that can be used to compute consistent quantile estimates for all sub-ranges in the prediction horizon; (iii) By learning from similar items, DeepAR is able to provide forecasts for items with little history, a case where traditional single-point forecasting methods fail; (iv) DeepAR does not assume Gaussian noise, but can incorporate a wide range of likelihood functions, allowing the user to choose one that is appropriate for the statistical properties of the data.

## Data

The recent works in economics and finance on the application of text analysis from social media and news generally suffer from a limited scope of historical financial news available, and from the limitation of the analysis to short texts only (e.g. usually tweets or news headlines, see e.g. [1, 8]). In our study we consider a long time period and analyse the entire text contained in the news articles. The source of economic news was obtained from a commercial provider^2^. The data set consists of several million articles, full-text, from January 1982 until September 2019 (approximately 40 years) for the following US outlets: The New York Times, The Wall Street Journal, The Washington Post, The Dallas Morning News, The San Francisco Chronicle, and the Chicago Sun-Times. The economic variable of interest is the spread between the yield on 10-year T-bonds and 3-month T-bill, denoted by T10Y3M, which is obtained from the Saint Louis Fed FRED repository^3^.

## Information Extraction from News

In this section we describe the NLP information extraction pipeline used to extract sentences from the news text that refer to specific economic aspects, which we then use to derive the Word Embedding to be used in the forecasting exercise. The method works as follow. Suppose we want to extract from our news data the embeddings referred to a specific economic concept, for instance *industrial production*. First, its economic synonyms are derived from SPARQL queries of the *World Bank Group Ontology*^4^. For example, for *industrial production*, the economic synonyms that are obtained are: manufacturing; industrial output; secondary sector; industry productivity; manufacturing development; industrial growth; manufacturing productivity; etc. Given the goal of forecasting the T10Y3M time series, we used search keywords that are broadly related to the economy and to monetary and fiscal policy (the complete list can be found in the Appendix).

Afterwards, we employ a rule-based procedure that builds on the linguistic features of the *spaCy* Python library^5^. The NLP pipeline relies on the *en_core_web_lg* model of spaCy^6^, an English multi-task Convolutional Neural Network trained on OntoNotes and GloVe vectors trained on Common Crawl. The role of *spaCy* is to provide structured information about the text analyzed in the form of word vectors, context-specific token vectors, Part-of-speech (POS) tags, dependency parse and named entities. The following NLP steps are performed:

- *Tokenization & lemmatization:* News text is split into meaningful segments (*tokens*), considering noninflected form of the words (*lemmas*) in the text taken from WordNet^7^.
- *Named Entity Recognition:* Named-entity mentions in the news text are located and classified, including locations, organizations, time expressions, quantities, monetary values, etc.
- *Most frequent location:* Heuristic procedure which assigns the location to which a sentence is referring, as its most frequent named-entity location detected in the sentence text.
- *POS tagging:* News text is parsed and tagged using spaCy’s statistical model. We loop over the part-of-speech tags, stopping when our search concept, or one of its synonyms, is found.
- *Dependency Parsing:* After our search concept is found in a sentence, we loop over the available dependency parsing tree of the sentence. In particular we navigate over the neighbouring tokens of the discovered search concept by employing a rule-based approach leveraging on the syntactic dependency parsing tree. In this way chunks of terms related to our search concept are constructed.
- *Tense detection:* Heuristic procedure used to detect the tense of the constructed terms chunks extracted from the news and related to our search concept through our rule-based approach.

After the NLP pipeline has produced the chunks of terms related to our search concepts, we merge the GloVe Word Embeddings by averaging each embedding features, thus producing a unique vector of embeddings representing the extracted terms in that sentence. Similarly, all the extracted embeddings of the same frequency period of the time series considered (daily in our case) are merged together producing a unique vector of embeddings for each period. These represent the signals ordered in time that will be used as covariates for the forecasting exercise in our T10Y3M case study.

**Fig. 1.**
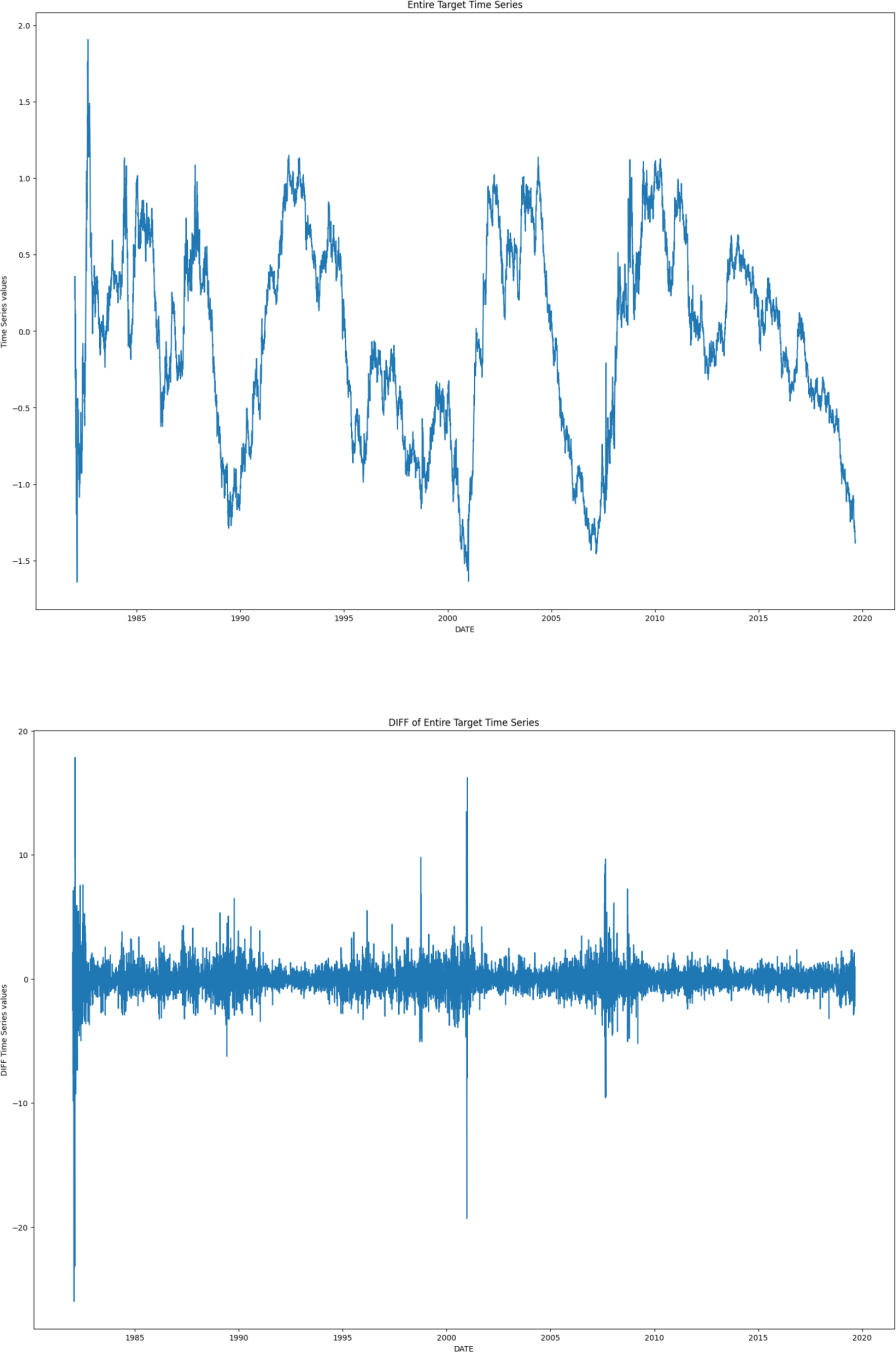
Time series of the yield spread between the US 10-Year Treasury Constant Maturity and the 3-Month Treasury Constant Maturity (T10Y3M; top panel), and the change of T10Y3M defined as the log-difference between consecutive observations (bottom panel).

## Preliminary Findings

In this section we show our preliminary findings on the application of DeepAR to the forecasting of the T10Y3M time series, augmented by the extracted Word Embedding from the US economic news. Given that the T10Y3M daily time series (see Fig. 1, top) is a highly persistent and non-stationary process, we take its first log-difference and obtain a stationary series of daily changes (see Fig. 1, bottom). Forecasting the yield spread differences is an extremely challenging task, as the series behaves similarly to a random walk process. The time ranges from January 1$^{st}$ 1982 to August 30$^{th}$ 2019. Missing values for the target time series and covariates, mainly related to weekends and holidays, are dropped from the analysis, giving a final number of 9, 418 data points for the whole target time series.

All training, validation, and test data are historical values that have been smoothed using a logarithmic transformation and scaled on training data only. We have opted for a robust scaling of the variables by using statistics robust to the presence of outliers. That is, we remove the median to each time series, and the data are scaled according to the quantile range between the first quartile and the third quartile. Data standardization is a common requirement in the estimation of many machine learning models. Typically this is done by removing the mean and scaling to unit variance. However, outliers can often influence the sample mean/variance negatively. In such cases, as in ours, the median and the inter-quartile range provide better results. Centering and scaling happen independently on each feature by computing the relevant statistics on the training sets. These pre-processing approaches have been used throughout the analysis due to better experimental results relative to other smoothing and scale transformations. We employ a rolling window for training and validation, with a window length equal to half of the full sample, that is 4, 709 data points. For each window, we make one step-ahead forecasts.

**Fig. 2.**
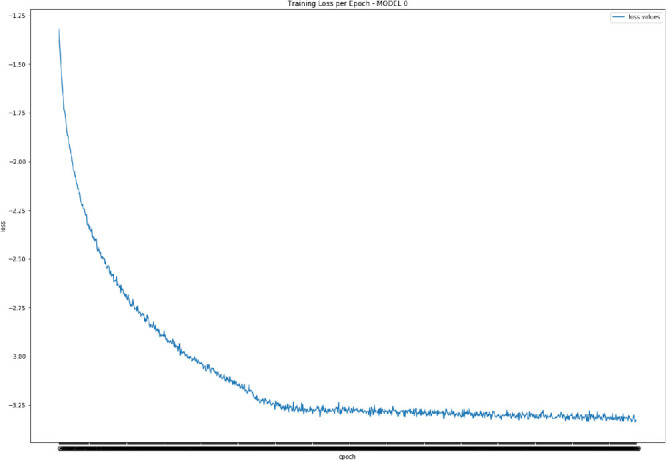
Example of negative log-likelihood training loss values for the first trained DeepAR model.

The DeepAR model is implemented by adopting Gluon Time Series (GluonTS) [2]^8^, an open-source library for probabilistic time series modelling that focuses on deep learning-based approaches. The library is available in Python and relies on Gluon, which is the joint AWS/Microsoft open-source deep learning solution interfacing Apache MXNet^9^. The DeepAR parameters are set experimentally to 2 RNN layers, each having 40 LSTM cells, and using a learning rate equal to 0.001. As mentioned earlier, the employed training loss is the negative log-likelihood, and the probability distribution to draw the probabilistic forecasts is the student *t*-distribution. We set also a re-training step for the model equal to 7 d, meaning that every 7 consecutive data points the DeepAR model is completely retrained. As concerned the number of epochs for each training, we choose experimentally the value of 500 epochs, which delivers convergence of the training loss (e.g., see in Fig. 2 the training loss values for the first trained model).

**Fig. 3.**
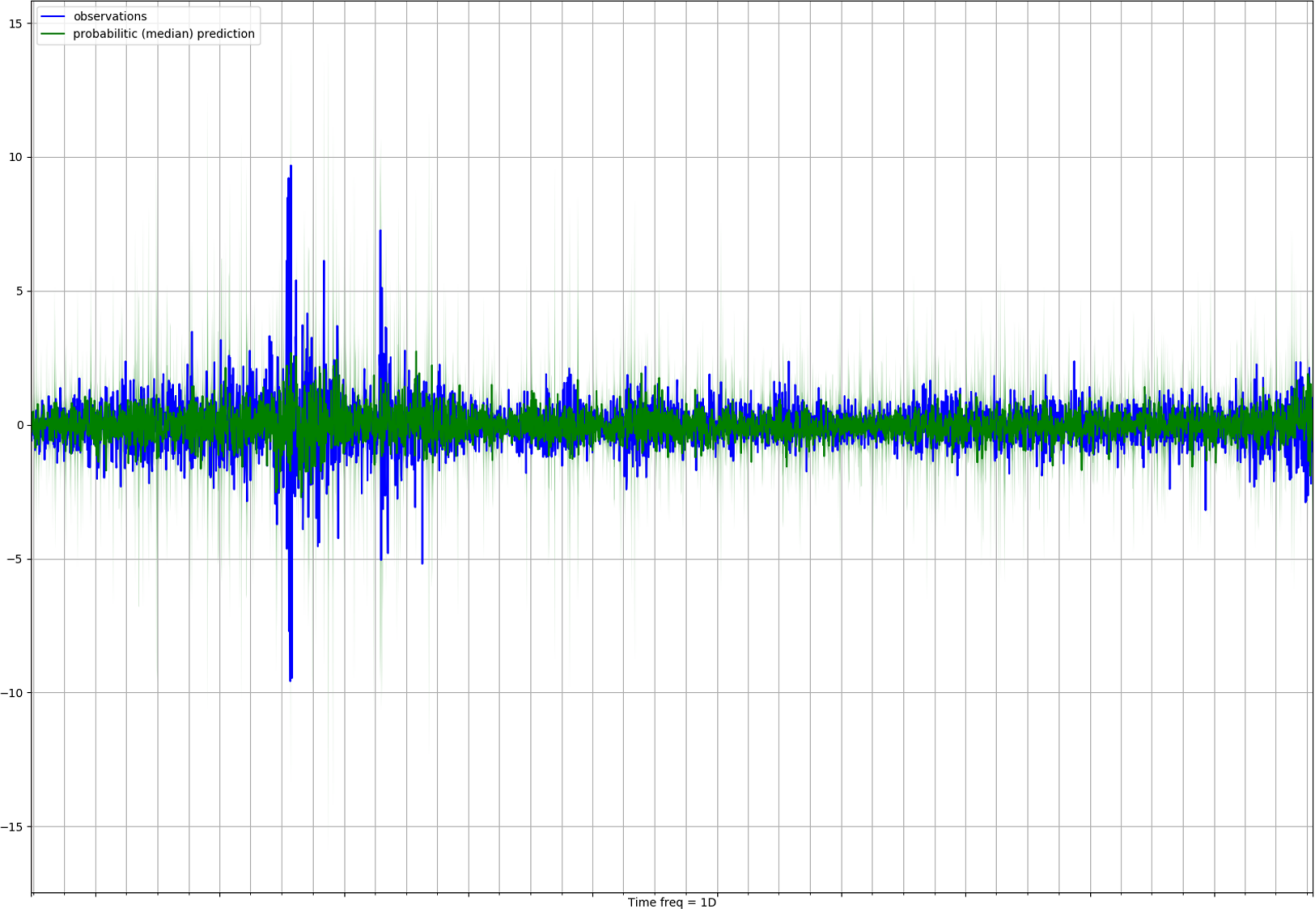
Median forecasts (green) and observations for the T10Y3M series (blue) for the entire forecasting period. (Color figure online)

The whole experiment is run in parallel on 40 cores at 2.10 GHz each into an Intel(R) Xeon(R) E7 64-bit server having overall 1 TB of shared RAM. The complete experiment required around 20 h of computation time.

**Fig. 4.**
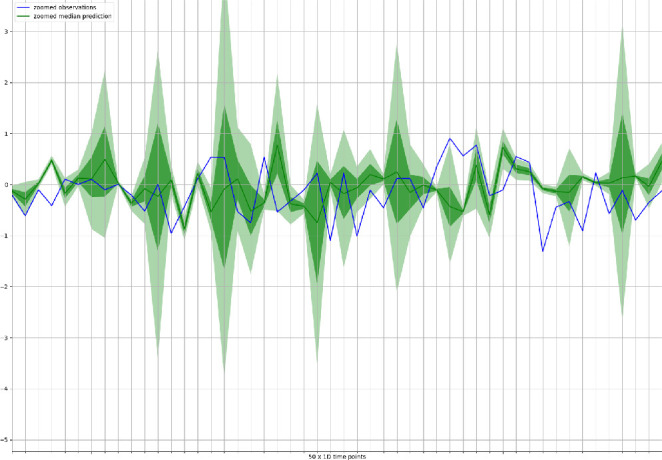
Probabilistic forecasts (green) and observations for the T10Y3M series (blue) for the first 50 d in the testing period. The green continuous line shows the median of the probabilistic predictions, while lighter green areas represents higher confidence intervals (dark green = 50% confidence interval; light green = 90% confidence interval”). (Color figure online)

Figure 3 shows the observations for the T10Y3M series (blue line) together with the median forecast (dark green line) and the confidence intervals in lighter green (i.e., dark green area = 50% confidence interval; light green area = 90% confidence interval"). To better visualize the differences between observed and predicted time series, we report the same plot on a smaller time range (50 d) in Fig. 4. A qualitative analysis of the figure suggests that the DeepAR forecasts do a reasonable job at capturing the variability and volatility of the time series. Forecasting an interval rather than a point is an important feature of the process since it provides an estimate of the uncertainty involved in the forecast which allows downstream decisions based to account for such uncertainty.

For a quantitative evaluation of the forecasts in the test set, we compute a number of commonly used metrics, such as the mean absolute scaled error (MASE), the symmetric mean absolute percentage error (sMAPE), the root mean square error (RMSE), and the (weighted) quantile losses (wQuantileLoss), that is the quantile negative log-likelihood loss weighted with the density. In particular, DeepAR obtains the following *in-sample results*:

- MASE = 0.42,
- sMAPE = 0.89,
- RMSE = 0.77,
- wQuantileLoss[0.1] = 0.24,
- wQuantileLoss[0.3] = 0.55,
- wQuantileLoss[0.5] = 0.65,
- wQuantileLoss[0.7] = 0.54,
- wQuantileLoss[0.9] = 0.24.

**Fig. 5.**
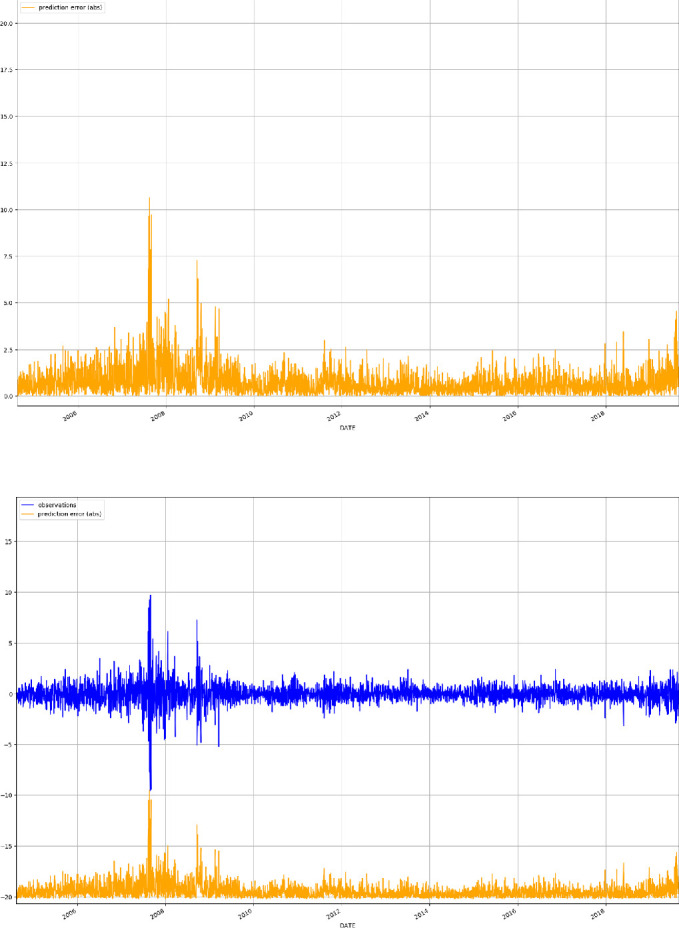
Median absolute forecast error (MAFE) (top panel), and real T10Y3M observations (blue) against MAFE (orange) (bottom panel). (Color figure online)

The *out-of-sample results* are instead:

- MASE = 0.75,
- sMAPE = 1.46,
- RMSE = 1.02,
- wQuantileLoss[0.1] = 0.82,
- wQuantileLoss[0.3] = 1.09,
- wQuantileLoss[0.5] = 1.16,
- wQuantileLoss[0.7] = 1.11,
- wQuantileLoss[0.9] = 0.84.

As expected the results worsen passing from the in-sample to the out-of-sample setting. However, the gap looks acceptable showing a good generalization of the trained model. Moreover, the model performs better at high (0.9) and low (0.1) quantiles, where it obtains lower weighted quantile losses. However, looking at Fig. 5, which shows the median absolute forecast error (MAFE, in orange) against the real T10Y3M observations (in blue), we see that the model is performing poorly during the crisis period (2007–2009), where the series presents clusters of high volatility. In future work, we aim to improve the performance of the algorithm by tweaking our NLP pipeline over the economic news by considering different economic searches and Word Embedding models, and by improving DeepAR’s forecasting results by changing architecture and/or hyperparameters.

To evaluate the forecasting performance of DeepAR we can compare the forecasting metrics against those produced by other models. For example, we can produce forecasts using a simple moving average (MA) and a naïve method (NM). With the moving average method, the forecasts of all future values are equal to the average (or “mean”) of the historical data. If we let the historical data be denoted by $y_1, ..., y_T$, then we can write the forecasts as $\hat{y}_{T+h|T} = \bar{y} = (y_1 + ... + y_T)/T$. The notation $\hat{y}_{T+h|T}$ is a short-hand for the estimate of $y_{T+h}$ based on the data $y_1, ..., y_T$. In our case we have chosen $T=7$, that is we do a one-week moving average on the T10Y3M time series. For the naïve forecasts, instead, we simply set all forecasts to be the value of the last observation for our target (i.e. the log-difference of T10Y3M). That is, $\hat{y}_{T+h|T}=\hat{y}_{T}$. Because naïve forecasts are optimal when data observations follow a random walk, these are also called random walk forecasts. The naïve method works well for many economic and financial time series, as is the case with our T10Y3M data. Table 1 reports the out-of-sample results for the three methods listed above. There is a clear superiority of the DeepAR algorithm with respect to the two other approaches. An extensive computational analysis where the algorithm will be compared with other state-of-the-art forecasting approaches is the goal of future work.

**Table 1.** Out-of-sample forecast evaluation for the *DeepAR*, *MA*, and *NM* models in terms of MASE, sMAPE, RMSE, and wQuantileLoss$_{mean}$ error metrics.

*metrics*	DeepAR	MA	NM
MASE	0.75	0.80	0.90
sMAPE	1.46	1.55	1.47
RMSE	1.02	1.04	1.30
wQuantileLoss$_{mean}$	0.88	0.93	1.39

## Conclusions

In this contribution we provide some early results of a currently on-going project aimed at exploring the predictive power of news for economic and financial time series forecasting. The novelties of the approach is that we use Word Embeddings as features which we use in the DeepAR neural forecasting method. In this initial experiment, we forecast the yield spread between the US 10-Year Treasury Constant Maturity and the 3-Month Treasury Constant Maturity (T10Y3M). The Word Embeddings are calculated based on the GloVe model and extracted from US economic news. After providing an overview of the methodology under current development, we report some preliminary results on this use-case application, showing satisfactory performance of the devised approach for such a challenging task. Information extracted from news looks relevant for the forecasting exercise of this economic variable.

Certainly further extensive research still needs to be done. We notice that DeepAR attains a poor forecasting performance during the crisis period (2007–2009), where the T10Y3M suffered from high volatility. We aim to improve the performance of the algorithm by tweaking our NLP pipeline over the economic news by considering different economic searches. Furthermore, while in this contribution we only use Glove Word Embedding, in the future we will perform a comparison with other models, like the popular Word2Vec and the recent context-dependent BERT model. Moreover, we will try to improve the performance of the implemented DeepAR model by changing architecture and optimizing its hyperparameters. Interpretability of the model by using, e.g., computed Shapley values, will be object of future investigation.

To conclude, our approach that combines Word Embedding and Deep Learning seems a promising direction to follow in order to improve the forecast accuracy of economic and financial time series. We believe that by combining these two methodologies, effective solutions can be built to improve effectiveness for this type of prediction tasks.

## Appendix: List of Keywords Used for the Information Extraction Pipeline

The considered single and compounded keywords were the ones belonging to the following two (lemmatized) terms sets:

- single terms:
  *“dividend, earning, nasdaq, bank, derivative, lending, borrowing, economy”.*
- compounded terms:
  *“financial market, corporate finance, stock market, capital market, currency market, derivative market, bond market, exchange market, stock price, s&p 500, dow jones, future trading, option trading, financial investor, dow jones industrial average, stock index, equity market, wall street, central bank, fomc, federal reserve, money supply, monetary policy, federal fund, base rate, interest rate, three-month treasury, threemonth treasury, ten-year treasury, ten year treasury, treasury yield, yield curve, yield spread, quantitative easing”.*

In addition, the considered search keywords contained also the pairwise combinations of the terms in the following two sets:

- single terms combination:
  *“banking, financial”.*
- compounded terms combination:
  *“sector, commercial, investment”.*

The locations and organizations used for restricting the searches to US articles only were:

- locations and organizations:
  *“america, united states, columbia, land of liberty, new world, u.s., u.s.a., us of a, usa, us, land of opportunity, the states, fed, federal reserve board, federal reserve, census bureau, bureau of economic analysis, treasury department, department of commerce, bureau of labor statistics, bureau of labour, department of labor, open market committee, bea, bureau of economic analysis, bis, bureau of statistics, board of governors, congressional budget office, cbo, internal revenue service, irs”.*

## Footnotes

1 In our case, we considered a student *t*-distribution in order to account for the fat-tail characteristic of financial returns.

2 Dow Jones DNA: Data, News and Analytics Platform: https://www.dowjones.com/dna/.

3 https://fred.stlouisfed.org/series/T10Y3M.

4 World Bank Group Ontology, available at: http://vocabulary.worldbank.org/thesaurus.html.

5 spaCy: Industrial-Strength Natural Language Processing. Available at: https://spacy.io/.

6 https://spacy.io/models/en.

7 WordNet, A Lexical Database for English. Available at https://wordnet.princeton.edu/.

8 Available at: https://gluon-ts.mxnet.io/#gluonts-probabilistic-time-series-modeling.

9 Available at: https://mxnet.apache.org/.
